# Detection and psychoprophylaxis: therapy through art in institutional contexts in cluj-napoca romania

**DOI:** 10.1192/j.eurpsy.2023.1734

**Published:** 2023-07-19

**Authors:** C. Emilia

**Affiliations:** Centru Comunitar Județean Complex Servicii Sociale Comunitare pentru Copii și Adulți Cluj, CONSILIUL JUDEȚEAN CLUJ DIRECŢIA GENERALĂ DE ASISTENŢĂ SOCIALĂ ŞI PROTECŢIA COPILULUI CLUJ, Cluj-Napoca, Romania

## Abstract

**Introduction:**

Considering the development of this study, we selected cases where art therapy played a central role in the educational/therapeutic process. Studied 130-150 cases per year on average, for 25 years (1996-2021), including children and adolescents aged between 2 and 18 years of both sexes, different social backgrounds in terms of housing, culture, and education: education/ clinical art therapy ( Mental Health Center for Children and Adolescents ward of the Cluj-Napoca Children’s Emergency Hospital) and non-clinical education/art-therapy.

The activities also have components of artistic creation, research, and teaching with students, within the disciplines “Art therapy in institutional contexts” and “Artistic play and experiment in group dynamics”, within the University of Art and Design section Pedagogy of Plastic and Decorative Arts from Cluj-Napoca Romania. The results are published at the international and world congresses to which we were invited together with the practitioners under supervision.

**Objectives:**

The aims of occupational therapy, which include art therapy and play therapy, are to facilitate the use of creative process and symbolic communication, associated with narrative and imitation, to develop new ways of communication, self-expression and seeing things.

**Methods:**

We use materials and techniques that are specific to visual arts (painting, sculpture, graphic, multimedia, photography, film, animation, and digital media), but also traditional ones, specific to tridimensional arts, such as pottery wheels and sculptural modeling. Activities are structured according to the following dimensions:

- The making of art or the production of other crafts resembles a situation test
- The analysis of the products allows the beneficiaries to attain a certain level of introspection and to “work through” their problems in constructive manner
- The execution of an operation requires sensory, cognitive, and affective intervention;
- Psychological dimensions, which include the individual’s intrinsic need for self-improvement, for obtaining competence and self-knowledge;
- The socio-cultural and symbolic dimension of the act;
- The spiritual dimension, related to the meaning of the occupation for the individual;
- The temporal dimensions of the occupation (referring to the time or period of time required for recovery).

**Results:**

By interacting with these factors, the individual gets to know his own potential and limits, but also those of the environment in which he lives.

**Image:**

**Figure:**
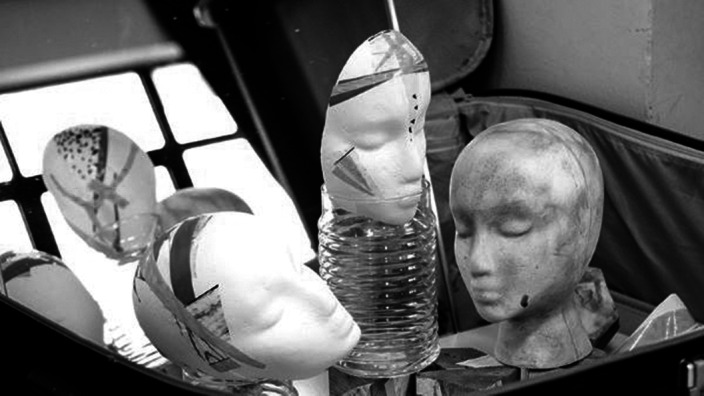

**Image 2:**

**Figure:**
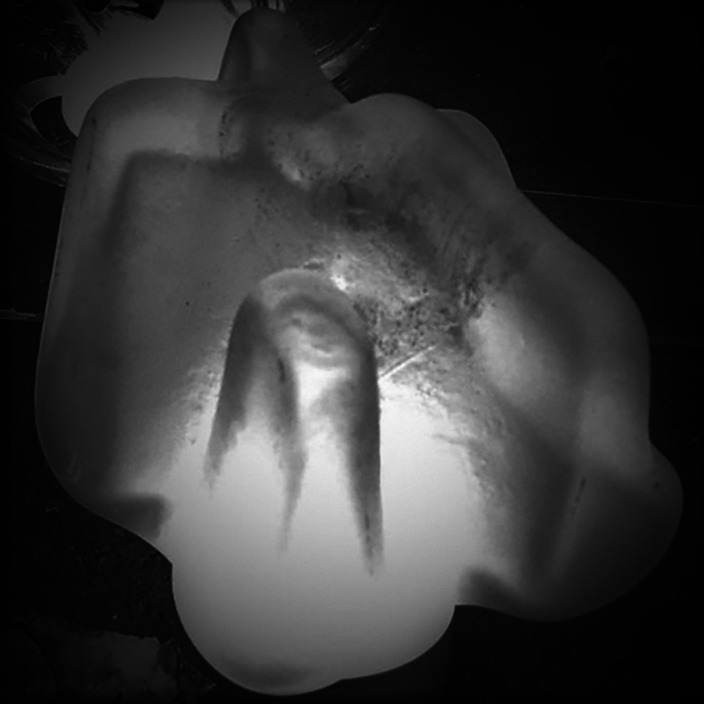

**Conclusions:**

An equidistant trialogue and circular relations between art, religion and science, without any specific supremacy, is created, which can offer from the start the possibility of lasting harmonizations, of informational transfers and professional enhancements that support developments, ennobling the human being through positive reorientations and beneficial recoveries.

**Disclosure of Interest:**

None Declared

